# (4-Fluoro­phen­yl)(2-hy­droxy-5-methyl­phen­yl)methanone

**DOI:** 10.1107/S1600536814001883

**Published:** 2014-02-12

**Authors:** C. S. Dileep, V. Lakshmi Ranganatha, N. K. Lokanath, S. A. Khanum, M. A. Sridhar

**Affiliations:** a Department of Studies in Physics, Manasagangotri, University of Mysore, Mysore 570 006, India; b Department of Chemistry, Yuvaraja’s College, University of Mysore, Mysore 570 005, India

## Abstract

In the title compound, C_14_H_11_FO_2_, the dihedral angles beteen the central C_3_O ketone residue and the fluoro- and hy­droxy-substituted benzene rings are 50.44 (9) and 12.63 (10)°, respectively. The planes of the benzene rings subtend a dihedral angle of 58.88 (9)° and an intra­molecular O—H⋯O hydrogen bond closes an *S*(6) ring. No directional inter­actions beyond van der Waals packing contacts were identified in the crystal structure.

## Related literature   

For related structures, see: Dileep *et al.* (2013 ▶); Mahendra *et al.* (2005 ▶).
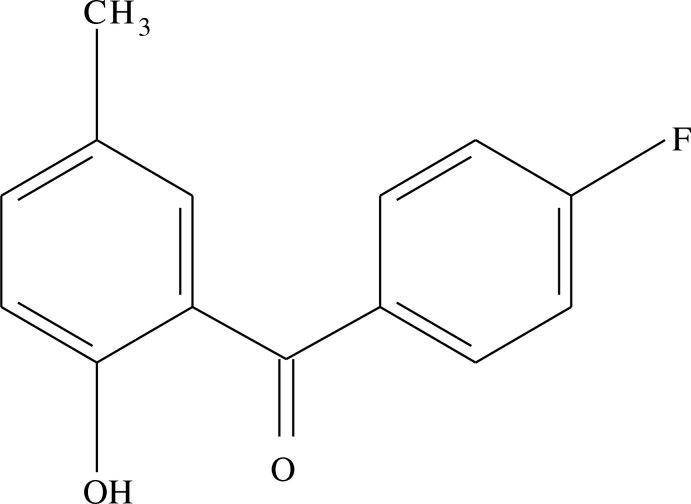


## Experimental   

### 

#### Crystal data  

C_14_H_11_FO_2_
*M* *_r_* = 230.23
Orthorhombic, 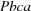

*a* = 5.9396 (6) Å
*b* = 12.3808 (15) Å
*c* = 30.522 (3) Å
*V* = 2244.5 (4) Å^3^
*Z* = 8
Cu *K*α radiation
μ = 0.85 mm^−1^
*T* = 296 K
0.28 × 0.25 × 0.22 mm

#### Data collection  

Bruker X8 Proteum CCD diffractometer
Absorption correction: multi-scan (*SADABS*; Bruker, 2013 ▶) *T*_min_ = 0.798, *T*_max_ = 0.836
8509 measured reflections
1822 independent reflections
1535 reflections with *I* > 2σ(*I*)
*R*_int_ = 0.030

#### Refinement  

*R*[*F* ^2^ > 2σ(*F* ^2^)] = 0.047
*wR*(*F* ^2^) = 0.135
*S* = 1.06
1822 reflections
156 parameters
H-atom parameters constrained
Δρ_max_ = 0.18 e Å^−3^
Δρ_min_ = −0.21 e Å^−3^

### 

Data collection: *APEX2* (Bruker, 2013 ▶); cell refinement: *SAINT* (Bruker, 2013 ▶); data reduction: *SAINT*; program(s) used to solve structure: *SHELXS97* (Sheldrick, 2008 ▶); program(s) used to refine structure: *SHELXL97* (Sheldrick, 2008 ▶); molecular graphics: *Mercury* (Macrae *et al.*, 2008 ▶); software used to prepare material for publication: *Mercury*.

## Supplementary Material

Crystal structure: contains datablock(s) global, I. DOI: 10.1107/S1600536814001883/hb7191sup1.cif

Structure factors: contains datablock(s) I. DOI: 10.1107/S1600536814001883/hb7191Isup2.hkl

Supporting information file. DOI: 10.1107/S1600536814001883/hb7191Isup3.cml

CCDC reference:

Additional supporting information:  crystallographic information; 3D view; checkCIF report

## Figures and Tables

**Table 1:** Hydrogen-bond geometry (Å, °)

*D*—H⋯*A*	*D*—H	H⋯*A*	*D*⋯*A*	*D*—H⋯*A*
O1—H1⋯O2	0.82	1.86	2.574 (2)	146

**Fig. 1.**
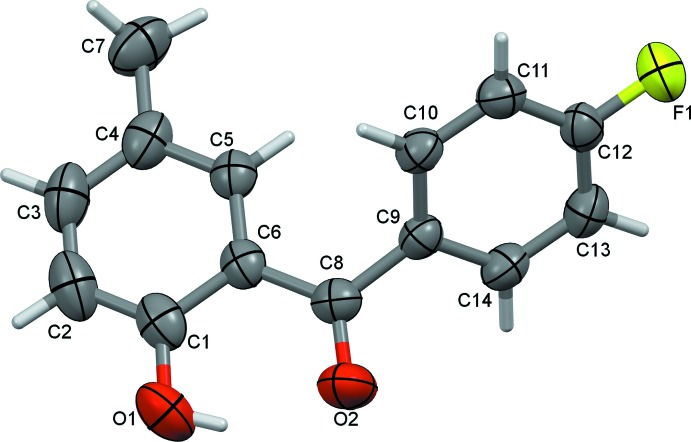
ORTEP view of the molecule with dispalcement ellipsoids drawn at the 50% probability level.

**Fig. 2.**
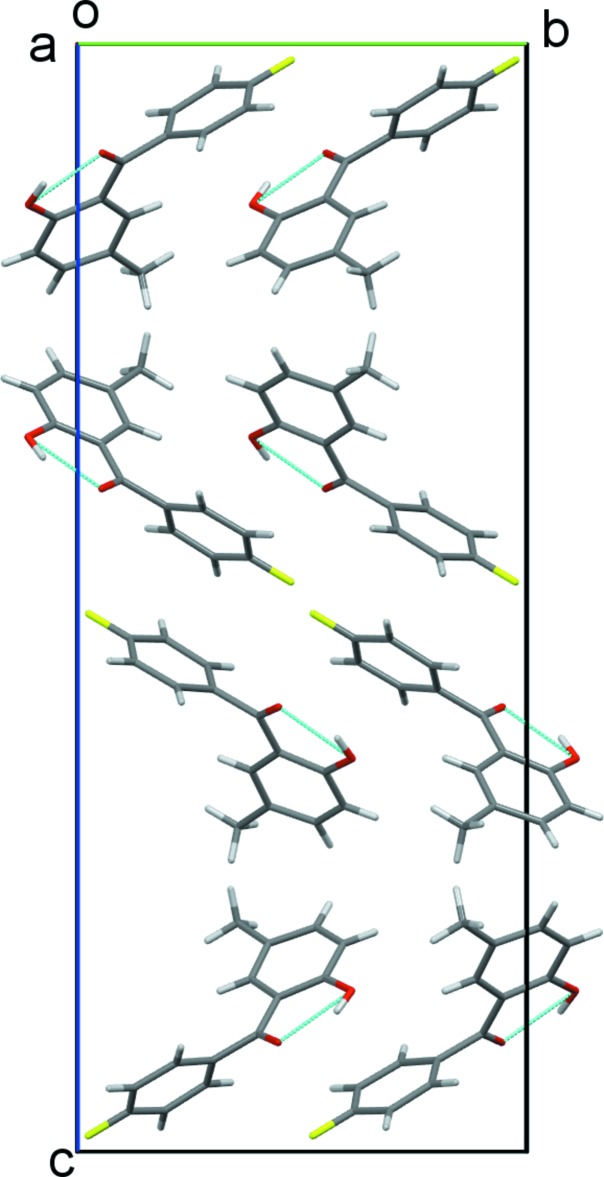
A molecular packing view of the title compound down the a-axis.

## supplementary crystallographic information

### 1. Comment

As part of our structural studies of benzophenone derivatives (Dileep *et al.*, 2013), the title compound was prepared and characterized by single-crystal X-ray diffraction.

The mean plane angle between the phenyl rings (/C1C2/C3/C4/C5/C6) and (C9/C10/C11/C12/C13/C14) is 58.88°.

The position of C8 atom is distorted trigonal planar geometry as indicated by bond angle values (O2—C8—C6)=121.05°, (O2—C8—C9) =118.37°, (N6—C8—C9)= 120.56°.

The conformation of the attachment of the two phenyl rings to the central carbonyl group can also be characterized by torsion angles (O2—C8—C6—C5) and (O2—C8—C9—C10) of -165.31° and -129.32°, respectively.

The crystal structure exhibits intramolecular O(1)—H(1)···O(2) hydrogen bonds. The bond angle between (O1—C1—C2) and(O1—C1—C6) is 118.61° and 122.22°. The bond length between (O1—C2) and (O2—C8) is 1.351 Å and 1.238 Å. The molecular packing when viewed down the *a* axis is shown in Fig. 2.

### 2. Experimental

A mixture of anhydrous aluminium chloride (0.03 mol) and 4-fluoro-benzoic acid *p*-tolyl ester (0.02 mol) in dry nitrobenzene (40 ml) was protected from moisture by calcium chloride guard tube and refluxed for 45 min. At the end of this period the solution was cooled and decomposed by acidulated ice-cold water. Nitrobenzene was removed by steam distillation. The residual solid was crushed into powder, dissolved in ether and extracted with 10% sodium hydroxide. The basic aqueous solution was neutralized with 10% hydrochloric acid. The filtered solid was washed with distilled water and recrystallized from ethanol solution to afford pale yellow blocks of the title compound.

### 3. Refinement

All H-atoms were positioned geometrically and refined using a riding model with C–H= 0.93–0.9600 Å and *U*_iso_(H)=1.2Ueq(C).

### Crystal data

**Table:**

C_14_H_11_FO_2_	*F*(000) = 960
*M**_r_* = 230.23	*D*_x_ = 1.363 Mg m^−^^3^
Orthorhombic, *P**b**c**a*	Cu *K*α radiation, λ = 1.54178 Å
Hall symbol: -P 2ac 2ab	Cell parameters from 8509 reflections
*a* = 5.9396 (6) Å	θ = 5.8–64.4°
*b* = 12.3808 (15) Å	µ = 0.85 mm^−^^1^
*c* = 30.522 (3) Å	*T* = 296 K
*V* = 2244.5 (4) Å^3^	Block, pale yellow
*Z* = 8	0.28 × 0.25 × 0.22 mm

### Data collection

**Table:**

Bruker X8 Proteum CCD diffractometer	1822 independent reflections
Radiation source: Bruker MicroStar microfocus rotating anode	1535 reflections with *I* > 2σ(*I*)
Helios multilayer optics monochromator	*R*_int_ = 0.030
Detector resolution: 10.7 pixels mm^-1^	θ_max_ = 64.4°, θ_min_ = 5.8°
φ and ω scans	*h* = −6→6
Absorption correction: multi-scan (*SADABS*; Bruker, 2013)	*k* = −14→8
*T*_min_ = 0.798, *T*_max_ = 0.836	*l* = −34→35
8509 measured reflections	

### Refinement

**Table:**

Refinement on *F*^2^	Secondary atom site location: difference Fourier map
Least-squares matrix: full	Hydrogen site location: inferred from neighbouring sites
*R*[*F*^2^ > 2σ(*F*^2^)] = 0.047	H-atom parameters constrained
*wR*(*F*^2^) = 0.135	*w* = 1/[σ^2^(*F*_o_^2^) + (0.0857*P*)^2^ + 0.3925*P*] where *P* = (*F*_o_^2^ + 2*F*_c_^2^)/3
*S* = 1.06	(Δ/σ)_max_ < 0.001
1822 reflections	Δρ_max_ = 0.18 e Å^−^^3^
156 parameters	Δρ_min_ = −0.21 e Å^−^^3^
0 restraints	Extinction correction: *SHELXL97* (Sheldrick, 2008), FC^*^=KFC[1+0.001XFC^2^Λ^3^/SIN(2Θ)]^-1/4^
Primary atom site location: structure-invariant direct methods	Extinction coefficient: 0.0018 (4)

### Special details

**Table:**

Geometry. Bond distances, angles *etc*. have been calculated using the rounded fractional coordinates. All su's are estimated from the variances of the (full) variance-covariance matrix. The cell e.s.d.'s are taken into account in the estimation of distances, angles and torsion angles
Refinement. Refinement on *F*^2^ for ALL reflections except those flagged by the user for potential systematic errors. Weighted *R*-factors *wR* and all goodnesses of fit *S* are based on *F*^2^, conventional *R*-factors *R* are based on *F*, with *F* set to zero for negative *F*^2^. The observed criterion of *F*^2^ > σ(*F*^2^) is used only for calculating -*R*-factor-obs *etc*. and is not relevant to the choice of reflections for refinement. *R*-factors based on *F*^2^ are statistically about twice as large as those based on *F*, and *R*-factors based on ALL data will be even larger.

### Fractional atomic coordinates and isotropic or equivalent isotropic displacement parameters (Å^2^)

**Table:**

	*x*	*y*	*z*	*U*_iso_*/*U*_eq_	
F1	0.2531 (2)	0.47588 (9)	0.01429 (4)	0.0737 (5)	
O1	−0.0006 (3)	−0.11052 (11)	0.14523 (5)	0.0738 (6)	
O2	−0.1159 (2)	0.05347 (12)	0.09849 (5)	0.0682 (5)	
C1	0.1798 (4)	−0.05074 (14)	0.15707 (6)	0.0552 (6)	
C2	0.3280 (4)	−0.09271 (16)	0.18766 (7)	0.0692 (8)	
C3	0.5047 (4)	−0.0321 (2)	0.20256 (6)	0.0699 (8)	
C4	0.5444 (3)	0.07352 (18)	0.18787 (6)	0.0576 (6)	
C5	0.4015 (3)	0.11264 (14)	0.15595 (5)	0.0471 (6)	
C6	0.2213 (3)	0.05270 (13)	0.13940 (5)	0.0453 (5)	
C7	0.7297 (4)	0.1418 (2)	0.20654 (7)	0.0776 (9)	
C8	0.0668 (3)	0.09745 (15)	0.10646 (6)	0.0469 (6)	
C9	0.1246 (3)	0.19838 (13)	0.08253 (5)	0.0414 (5)	
C10	0.3293 (3)	0.21172 (14)	0.06101 (5)	0.0455 (5)	
C11	0.3715 (3)	0.30463 (15)	0.03721 (6)	0.0481 (6)	
C12	0.2094 (3)	0.38360 (14)	0.03669 (5)	0.0482 (6)	
C13	0.0073 (3)	0.37430 (16)	0.05775 (6)	0.0537 (6)	
C14	−0.0356 (3)	0.27965 (15)	0.08026 (6)	0.0492 (6)	
H1	−0.07480	−0.07740	0.12700	0.1110*	
H2	0.30740	−0.16250	0.19810	0.0830*	
H3	0.60200	−0.06200	0.22310	0.0840*	
H5	0.42620	0.18170	0.14500	0.0560*	
H7A	0.73900	0.20840	0.19050	0.1160*	
H7B	0.87010	0.10380	0.20420	0.1160*	
H7C	0.69850	0.15690	0.23680	0.1160*	
H10	0.43820	0.15790	0.06260	0.0550*	
H11	0.50590	0.31330	0.02200	0.0580*	
H13	−0.09800	0.42990	0.05700	0.0640*	
H14	−0.17390	0.27030	0.09410	0.0590*	

### Atomic displacement parameters (Å^2^)

**Table:**

	*U*^11^	*U*^22^	*U*^33^	*U*^12^	*U*^13^	*U*^23^
F1	0.0800 (9)	0.0591 (7)	0.0819 (8)	−0.0030 (6)	−0.0052 (6)	0.0248 (6)
O1	0.0938 (12)	0.0487 (8)	0.0789 (10)	−0.0156 (8)	0.0071 (8)	0.0023 (7)
O2	0.0575 (9)	0.0700 (9)	0.0770 (10)	−0.0208 (7)	−0.0081 (7)	0.0058 (7)
C1	0.0713 (13)	0.0429 (9)	0.0513 (10)	0.0047 (9)	0.0138 (9)	−0.0033 (8)
C2	0.1017 (18)	0.0495 (11)	0.0564 (11)	0.0150 (12)	0.0105 (12)	0.0081 (9)
C3	0.0838 (16)	0.0755 (15)	0.0504 (11)	0.0266 (13)	−0.0023 (10)	0.0123 (10)
C4	0.0560 (11)	0.0728 (12)	0.0440 (9)	0.0156 (10)	0.0005 (8)	−0.0009 (9)
C5	0.0489 (10)	0.0486 (10)	0.0437 (9)	0.0066 (8)	0.0050 (7)	0.0007 (7)
C6	0.0500 (10)	0.0418 (9)	0.0441 (9)	0.0054 (8)	0.0074 (7)	−0.0017 (7)
C7	0.0625 (13)	0.1073 (19)	0.0630 (13)	0.0040 (13)	−0.0148 (10)	−0.0016 (12)
C8	0.0427 (10)	0.0482 (10)	0.0499 (10)	−0.0023 (8)	0.0017 (7)	−0.0052 (7)
C9	0.0385 (9)	0.0446 (9)	0.0410 (8)	0.0014 (7)	−0.0049 (6)	−0.0032 (6)
C10	0.0397 (9)	0.0460 (9)	0.0507 (9)	0.0049 (7)	−0.0016 (7)	−0.0032 (7)
C11	0.0431 (10)	0.0555 (10)	0.0458 (9)	−0.0032 (8)	0.0002 (7)	−0.0001 (7)
C12	0.0533 (11)	0.0448 (9)	0.0464 (9)	−0.0029 (8)	−0.0095 (8)	0.0055 (7)
C13	0.0511 (11)	0.0516 (10)	0.0584 (11)	0.0127 (9)	−0.0062 (8)	0.0022 (8)
C14	0.0375 (9)	0.0591 (11)	0.0510 (10)	0.0048 (8)	−0.0015 (7)	−0.0009 (8)

### Geometric parameters (Å, º)

**Table:**

F1—C12	1.357 (2)	C10—C11	1.383 (3)
O1—C1	1.352 (3)	C11—C12	1.372 (3)
O2—C8	1.238 (2)	C12—C13	1.367 (2)
O1—H1	0.8200	C13—C14	1.382 (3)
C1—C6	1.411 (2)	C2—H2	0.9300
C1—C2	1.384 (3)	C3—H3	0.9300
C2—C3	1.368 (3)	C5—H5	0.9300
C3—C4	1.402 (3)	C7—H7A	0.9600
C4—C5	1.380 (2)	C7—H7B	0.9600
C4—C7	1.500 (3)	C7—H7C	0.9600
C5—C6	1.397 (2)	C10—H10	0.9300
C6—C8	1.470 (2)	C11—H11	0.9300
C8—C9	1.488 (2)	C13—H13	0.9300
C9—C14	1.387 (2)	C14—H14	0.9300
C9—C10	1.392 (2)		
			
C1—O1—H1	110.00	C11—C12—C13	123.43 (17)
O1—C1—C6	122.23 (18)	C12—C13—C14	117.85 (17)
C2—C1—C6	119.16 (19)	C9—C14—C13	120.90 (16)
O1—C1—C2	118.61 (17)	C1—C2—H2	120.00
C1—C2—C3	120.41 (19)	C3—C2—H2	120.00
C2—C3—C4	122.29 (19)	C2—C3—H3	119.00
C3—C4—C5	116.72 (18)	C4—C3—H3	119.00
C3—C4—C7	121.83 (18)	C4—C5—H5	119.00
C5—C4—C7	121.44 (19)	C6—C5—H5	119.00
C4—C5—C6	122.69 (17)	C4—C7—H7A	109.00
C1—C6—C5	118.54 (16)	C4—C7—H7B	109.00
C5—C6—C8	121.70 (15)	C4—C7—H7C	109.00
C1—C6—C8	119.63 (16)	H7A—C7—H7B	109.00
O2—C8—C9	118.37 (16)	H7A—C7—H7C	110.00
C6—C8—C9	120.56 (15)	H7B—C7—H7C	110.00
O2—C8—C6	121.05 (17)	C9—C10—H10	120.00
C8—C9—C14	118.41 (16)	C11—C10—H10	120.00
C10—C9—C14	119.33 (15)	C10—C11—H11	121.00
C8—C9—C10	122.21 (15)	C12—C11—H11	121.00
C9—C10—C11	120.32 (16)	C12—C13—H13	121.00
C10—C11—C12	118.12 (16)	C14—C13—H13	121.00
F1—C12—C11	118.14 (15)	C9—C14—H14	120.00
F1—C12—C13	118.43 (16)	C13—C14—H14	120.00
			
O1—C1—C2—C3	−175.88 (19)	C5—C6—C8—C9	13.4 (3)
C6—C1—C2—C3	4.0 (3)	O2—C8—C9—C10	−129.33 (19)
O1—C1—C6—C5	174.98 (17)	O2—C8—C9—C14	48.0 (2)
O1—C1—C6—C8	−1.0 (3)	C6—C8—C9—C10	52.0 (2)
C2—C1—C6—C5	−4.9 (3)	C6—C8—C9—C14	−130.74 (18)
C2—C1—C6—C8	179.17 (18)	C8—C9—C10—C11	176.58 (16)
C1—C2—C3—C4	−0.1 (3)	C14—C9—C10—C11	−0.7 (2)
C2—C3—C4—C5	−2.7 (3)	C8—C9—C14—C13	−178.76 (17)
C2—C3—C4—C7	176.0 (2)	C10—C9—C14—C13	−1.4 (3)
C3—C4—C5—C6	1.7 (3)	C9—C10—C11—C12	2.0 (3)
C7—C4—C5—C6	−177.01 (17)	C10—C11—C12—F1	178.33 (15)
C4—C5—C6—C1	2.0 (3)	C10—C11—C12—C13	−1.3 (3)
C4—C5—C6—C8	177.92 (17)	F1—C12—C13—C14	179.67 (16)
C1—C6—C8—O2	10.5 (3)	C11—C12—C13—C14	−0.7 (3)
C1—C6—C8—C9	−170.79 (16)	C12—C13—C14—C9	2.1 (3)
C5—C6—C8—O2	−165.31 (17)		

### Hydrogen-bond geometry (Å, º)

**Table:**

*D*—H···*A*	*D*—H	H···*A*	*D*···*A*	*D*—H···*A*
O1—H1···O2	0.82	1.86	2.574 (2)	146
